# Environmentally robust *cis*-regulatory changes underlie rapid climatic adaptation

**DOI:** 10.1073/pnas.2214614120

**Published:** 2023-09-19

**Authors:** Mallory A. Ballinger, Katya L. Mack, Sylvia M. Durkin, Eric A. Riddell, Michael W. Nachman

**Affiliations:** ^a^Museum of Vertebrate Zoology, University of California, Berkeley, CA 94720; ^b^Department of Integrative Biology, University of California, Berkeley, CA 94720; ^c^Department of Biology, Utah State University, Logan, UT 84322; ^d^Department of Biology, Stanford University, Stanford, CA 94305; ^e^Department of Ecology, Evolution, and Organismal Biology, Iowa State University, Ames, IA 50011

**Keywords:** adaptation, *cis*-regulatory evolution, *plasticity*-eQTL, *Mus*

## Abstract

Changes in gene expression are thought to play a major role in adaptive evolution. While it is known that gene expression is highly sensitive to the environment, very few studies have determined the influence of genetic and environmental effects on adaptive gene expression differences in natural populations. Here, we utilize allele-specific expression to characterize *cis* and *trans* gene regulatory divergence in temperate and tropical house mice in two metabolic tissues under two thermal conditions. First, we show that gene expression divergence is pervasive between populations and across thermal conditions, with roughly 5 to 10% of genes exhibiting genotype-by-environment interactions. Second, we found that most expression divergence was due to *cis*-regulatory changes that were stable across temperatures. In contrast, patterns of expression plasticity were largely attributable to *trans*-effects, which showed greater sensitivity to temperature. Nonetheless, we found a small subset of temperature-dependent *cis*-regulatory changes, thereby identifying loci underlying expression plasticity. Finally, we performed scans for selection in wild house mice to identify genomic signatures of rapid adaptation. Genomic outliers were enriched in genes with evidence for *cis*-regulatory divergence. Notably, these genes were associated with phenotypes that affected body weight and metabolism, suggesting that *cis-*regulatory changes are a possible mechanism for adaptive body size evolution between populations. Our results show that gene expression plasticity, largely controlled in *trans*, may facilitate the colonization of new environments, but that evolved changes in gene expression are largely controlled in *cis*, illustrating the genetic and nongenetic mechanisms underlying the establishment of populations in new environments.

## Significance

Gene expression variation is shaped by both genetic and environmental effects, yet these two factors are rarely considered together in the context of adaptive evolution. We studied environmental influences on gene regulatory evolution in temperate and tropical house mice in cold and warm laboratory environments. We found that genetic effects in the form of *cis*-regulatory divergence were pervasive and largely insensitive to the environment. Many of these genetic effects are under selection and are associated with genes that affect body size, suggesting *cis*-regulatory changes as a possible mechanism for adaptive body size evolution. We also found many *trans*-effects controlling expression plasticity, demonstrating the importance of both genetic and nongenetic changes associated with adaptation over short timescales (a few hundred generations).

A major goal in evolutionary biology is to understand how organisms adapt to novel environments. Changes in gene expression have long been recognized to play a significant role in adaptive evolution (1, 2), especially across short evolutionary timescales (3, 4). Gene expression is highly sensitive to the environment, and genotype-by-environment interactions (GxE) constitute a large proportion of gene expression variation (5–8). Moreover, selection on genetic variation underlying plasticity may facilitate the colonization of new environments, especially during the initial stages of adaptation (9–11). Yet, we have a relatively poor understanding of how expression plasticity is controlled and how regulatory architecture evolves in populations adapting to different environments. For instance, while numerous studies have supported the evolution of gene expression through *cis*-regulatory changes (e.g., mutations in promoters and enhancers) (12–16), the extent to which these changes are environmentally sensitive and modulate expression plasticity is not well understood. *Trans*-effects (e.g., transcription factors) may also play a significant role in plastic changes in gene expression by modifying gene regulatory networks. Selection may then favor divergence through *trans*-acting mechanisms when such changes are beneficial in new environments (17, 18). Determining how both genetic and environmental effects influence the evolution of gene expression differences in natural populations is key to understanding the molecular mechanisms of adaptation.

The recent expansion of house mice into the Americas provides an opportunity to address the environmental sensitivity of gene regulatory changes involved in adaptive evolution. Since their arrival from Western Europe ~500 y ago, house mice [*Mus musculus domesticus* (M.m.d.)] have rapidly adapted to various climatic extremes through changes in morphology, physiology, and behavior (19–23). One striking example of this is changes in body size, as mice from more northern populations are significantly larger than mice closer to the equator, likely reflecting adaptation to differing thermal environments (23). Previous studies point to an important role for gene regulation in driving this local adaptation. First, genomic scans have primarily identified positive selection on noncoding regions (20, 21), which have been linked to differences in gene expression (21, 24). Second, changes in *cis-*regulation at specific loci have been associated with variation in body weight in North American mice (24). Finally, gene expression plasticity has been shown to differ between populations in response to environmental stressors (22), suggesting a role for environment-specific regulatory divergence in local adaptation.

Here, we investigate the role of gene regulation in the rapid adaptation of house mice to contrasting thermal environments. Specifically, using RNA-seq data collected from liver and brown adipose tissue (BAT) in males and females, we measured gene expression divergence in inbred lines of temperate and tropical mice and in their F1 hybrids when reared under warm and cold temperatures. This allowed us to describe the proportion of divergently expressed genes that are due to changes in *cis*, *trans*, or both and to determine the degree to which *cis*- and *trans*-regulation is temperature-dependent. Finally, we performed scans for selection in wild populations of house mice to identify genomic signatures of adaptation. We then intersected these genomic outliers with genes exhibiting *cis*-regulatory divergence to identify *cis*-regulatory mutations associated with local adaptation. Our results provide insight into how gene regulation changes in response to the environment and how complex regulatory divergence within a species may contribute to adaptive evolution.

## Results

### Extensive Gene Expression Divergence between Temperate and Tropical House Mice.

**Fig. 1.**
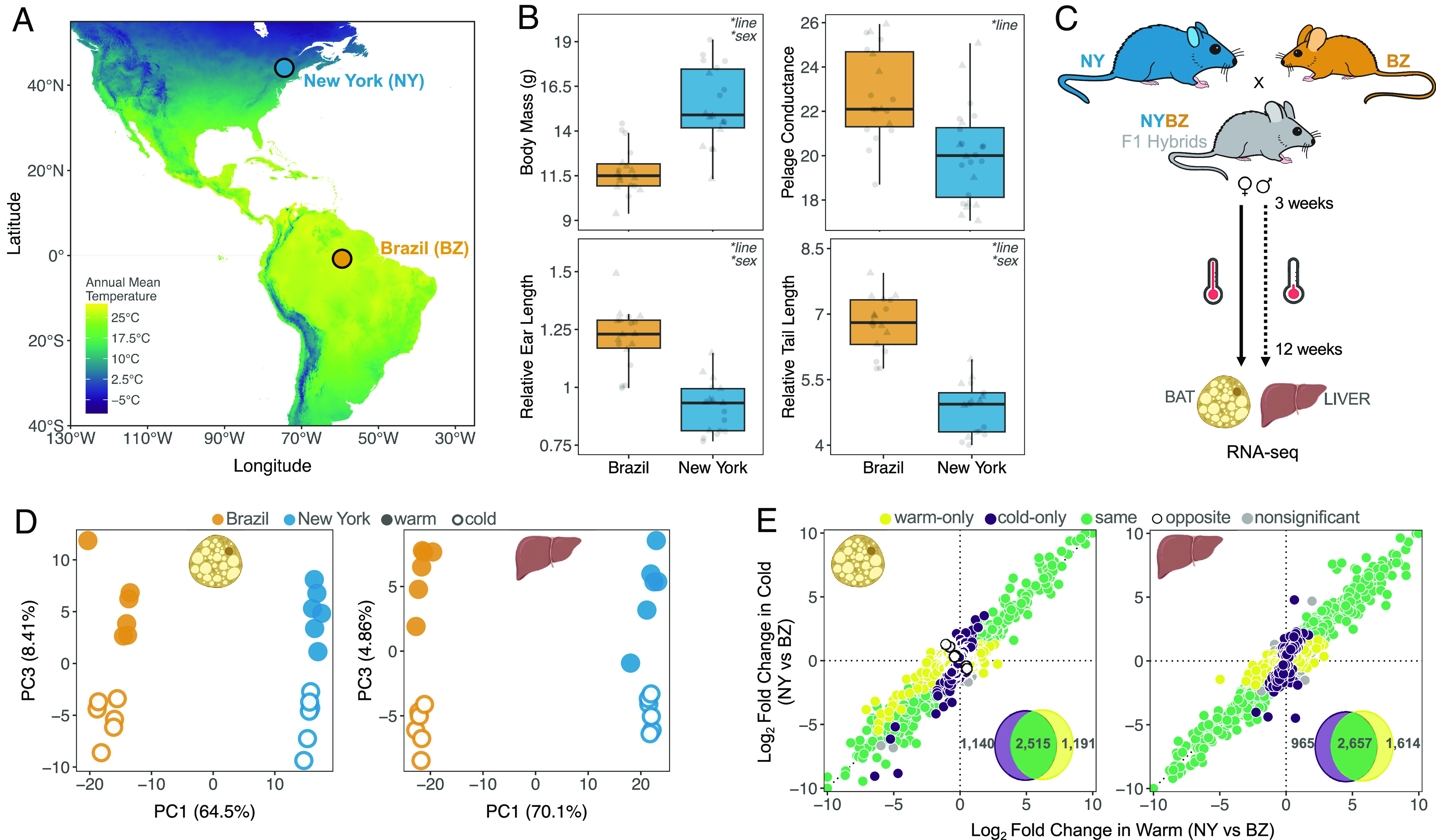
Evolved differences in phenotypes and gene expression. (*A*) Variation in mean annual temperature across North and South America. Wild-derived inbred lines originate from upstate New York (43°N) and equatorial Brazil (3°S). (*B*) Differences in body mass (g), pelage conductance (W m^−2^ °C^−1^), tail length (mm), and ear length (mm) between wild-derived inbred lines of New York (SARA) and Brazil (MANA). Tail length and ear length are plotted relative to body mass for each individual. Individuals are represented as individual points, and boxplots indicate the 25th, median, and 75th quartiles. Results from linear mixed models are presented in upper right corners (*P < 0.05; *SI Appendix*, Table S1). Males (circles) and females (triangles) show similar patterns and are combined for plotting simplicity. (*C*) Common garden experimental design. Individuals were reared under two temperatures from weaning until adults. (*D*) Principal component plots for PC1 vs. PC3 based on male gene expression in BAT and liver. PC1 separates individuals based on genotype while PC3 reflects environmental differences. Principal component plots for PC1 vs. PC2 are provided in *SI Appendix*, Figs. S1 and S2. (*E*) Expression divergence between New York and Brazil males in warm and cold for both BAT and liver. Log2 fold changes between parents were calculated for all genes independently. In each panel, points (representing individual genes) are colored depending on their direction and significance of the log2 fold change. *Insets* depict the total number of differentially expressed genes for each comparison (FDR < 0.05). Females show similar patterns and are depicted in *SI Appendix*, Figs. S2 and S3.

To characterize the regulatory architecture of adaptation, we first examined gene expression differences in two wild-derived inbred lines of house mice from different environments in the Americas: Saratoga Springs, New York, USA (SARA), located at 43°N, and Manaus, Amazonas, Brazil (MANA), located near the equator at 3°S. Saratoga Springs and Manaus differ considerably in climate, such as mean annual temperature (Fig. 1*A*), and mice from these environments show population-level differences in various traits, including morphology and gene expression (21, 23) (*SI Appendix, Supplementary Text*). Specifically, mice from New York are larger, retain more heat through their fur, and have shortened extremities compared to mice from Brazil (ANOVA tests, *P* < 0.05) (Fig. 1*B* and *SI Appendix*, Table S1), suggesting adaptation to different climates (23).

We explored patterns of gene expression evolution by rearing inbred lines from New York and Brazil under two temperatures (5 °C and 21 °C) and sequenced BAT and liver transcriptomes of 48 individuals (6/line/sex/environment) (Fig. 1*C*). We chose these two tissues as they play important roles in both metabolism and adaptive thermogenesis (25–27). Principal component analysis (PCA) of all gene expression data revealed tissue type as the largest source of variance (PC1 ~97% of variance explained), followed by sex (PC2 ~ 1.5%) (*SI Appendix*, Fig. S1). Within each tissue and sex, New York and Brazil mice cleanly separated along PC1 (>60% of variance explained), while PC3 largely separated warm- and cold-reared mice (>4% of variance explained) (Fig. 1*D* and *SI Appendix*, Fig. S2). We also identified more than a third of genes to be differentially expressed between New York and Brazil mice [false discovery rate (FDR) < 0.05] (Fig. 1*E* and *SI Appendix*, Figs. S3 and S4), with most expression differences concordant across environments and sexes.

**Fig. 2.**
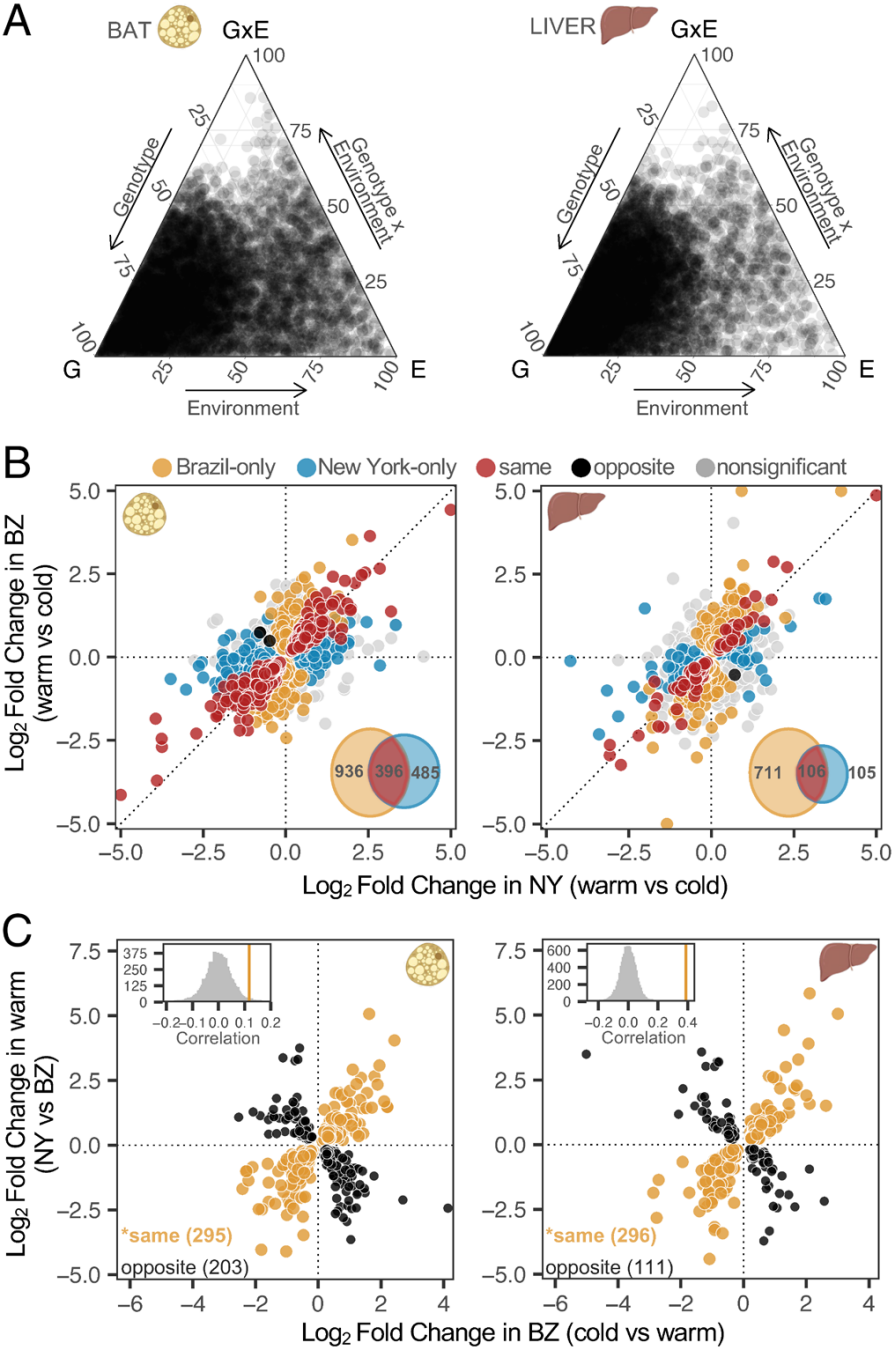
Patterns of genotype-by-environment interactions (GxE). (*A*) Ternary plots depicting the proportion of each gene’s expression variance explained by genotype (G), environment (E), and GxE. The relative proportion of each factor is shown for all differentially expressed male genes in BAT and liver. Total variance is the sum of all three components. (*B*) Comparison of gene expression differences between temperature regimes in NY and BZ males in both tissues. Log2 fold changes between temperatures were calculated for all genes independently. In each panel, points (representing individual genes) are colored depending on their direction and significance of the log2 fold change. GxE categories include line-specific responses or opposite responses between lines (*Materials and Methods*). *Insets* depict the total number of differentially expressed genes for each comparison (FDR < 0.05). (*C*) The relationship between gene expression plasticity and evolved divergence in both tissues. Points represent expression differences with statistically significant plasticity in BZ (cold vs. warm; FDR < 0.05) as well as significant expression divergence between NY and BZ at warm temperature (FDR < 0.05). Points colored in orange represent genes with a positive correlation between plasticity and evolved divergence, while points in black represent genes with a negative correlation. *Insets* depict that the observed correlation coefficient (orange solid lines) is more positive than a randomized distribution of correlation coefficients for each tissue (*Materials and Methods*). Asterisks denote significance of plasticity for each tissue (binomial exact tests, *P* < 0.05). Females show similar patterns and are depicted in *SI Appendix*, Figs. S2 and S3.

This strong pattern of divergence between lines was also apparent when we categorized differentially expressed genes as those showing genetic variation (G), environmental variation [i.e., plasticity (E)], or genetic variation for plasticity (i.e., GxE) (Fig. 2*A* and *SI Appendix*, Fig. S3). Genotype had >1.5× larger effect size (calculated as the mean absolute value of the log2 fold change) on differential gene expression than environment across both tissues (Fig. 2*A* and *SI Appendix*, Fig. S3). Similar effects were identified when we attributed expression differences to genotype and sex, although these patterns were largely tissue-dependent (*SI Appendix*, Fig. S4). Overall, these results demonstrate that within sexes and tissues, genotype plays a larger role than either environment or GxE in shaping expression differences.

### Reduced Gene Expression Plasticity in Cold-Adapted Mice.

Given that New York and Brazil mice have evolved under different thermal environments, we reasoned that gene expression responses to temperature would differ between these lines. Roughly ~5% and ~10% of all expressed genes showed significant GxE in liver and BAT, respectively (FDR < 0.05) (Fig. 2*B* and *SI Appendix,* Fig. S3 and Table S2). Although 3 genes showed opposite responses between lines across BAT (*cyfip2*, *wnt11*) and liver (*cmpk2*), most GxE patterns were categorized as line-specific in both tissues. Notably, we found fewer differentially expressed genes between environmental conditions in New York mice (~5% BAT; ~1% liver) than Brazil mice (~10% BAT; ~5% liver) (Chi-square tests, liver and BAT: *P* < 0.05), suggesting that New York mice may be less sensitive to cold stress.

Next, we explored the relationship between gene expression plasticity and evolved gene expression differences. Plasticity may facilitate the colonization of new environments by moving a population closer to the phenotypic optimum or, alternatively, reduce fitness under new environmental stressors (10, 28). To determine whether the pronounced transcriptional response to temperature of Brazil mice aligns with expression divergence between lines (*sensu* refs. 29 and 30), we asked whether the direction of expression plasticity of Brazil mice correlates with total expression divergence between New York and Brazil mice (*Materials and Methods*). We found that expression plasticity generally goes in the same direction as evolved divergence (positive Spearman’s correlations in both tissues, *P* < 0.05) (Fig. 2*C* and *SI Appendix*, Fig. S3), consistent with the idea that this plasticity is adaptive (22, 29, 31, 32). However, this pattern was less prominent in BAT, with only slightly more genes exhibiting concordant than discordant expression patterns (Fig. 2*C*).

### Expression Divergence Is Predominantly Due to Cis-Regulatory Changes, Which Are Enriched for Body Size and Metabolism.

**Fig. 3.**
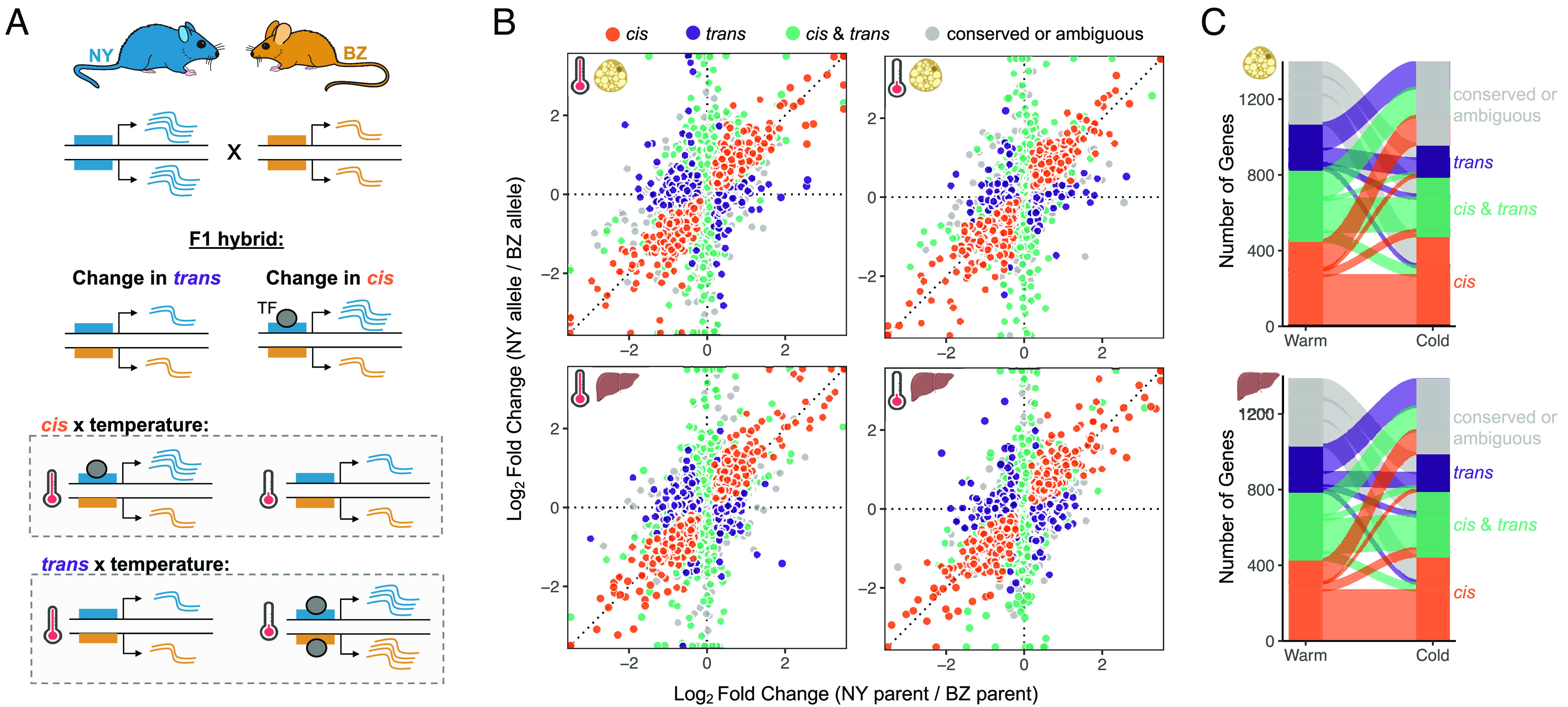
The relative distribution of regulatory changes between New York and Brazil house mice across environments. (*A*) Schematic depicting how *cis*- and *trans*-changes can be inferred with F1 hybrids, and how environmental differences may result in *cis* x temperature and *trans* x temperature effects. Blue and gold boxes represent *cis*-regulatory regions for NY and BZ, respectively. Wavy lines depict transcript levels of an allele. TF = transcription factor. (*B*) Categorization of regulatory divergence by comparing the expression of NY and BZ parents to NY- and BZ allele–specific expressions within F1s. Points (individual genes) represent log2 fold changes between reads mapping to each allele in the hybrid (BZ allele/NY allele; *y* axis) and the reads mapping to each parental line (BZ parent/NY parent; *x* axis). Genes are colored based on their inferred regulatory category: orange = *cis*, purple = *trans*, green = *cis* and *trans*, and gray = conserved or ambiguous. Genes categorized as conserved or ambiguous (gray points) constitute roughly 75% of all genes and are centered on the origin and mostly hidden behind other genes. (*C*) Changes in the number of genes for each inferred regulatory category between temperature regimes are illustrated in the alluvial plot. Genes that were conserved or ambiguous (gray) at both temperatures (~75%) are depicted in *SI Appendix*, Fig. S7.

To investigate the gene regulatory mechanisms underlying expression differences between New York and Brazil mice, we generated BAT and liver RNA-seq from NY x BZ F1 hybrids reared in both warm and cold environments (Fig. 1*C* and *SI Appendix*, Fig. S5). Measuring gene expression in F1 hybrids allowed us to discern whether parental gene expression differences are due to *cis*- and/or *trans*-acting changes by assessing patterns of allele-specific expression (ASE) (Fig. 3*A*). Specifically, as F1 hybrids inherit both a Brazil allele and New York allele within the same *trans*-acting environment, differences in expression between alleles are indicative of one or more *cis*-acting elements (33–36). In contrast, if no ASE is detected in hybrids but differences are observed between parental lines, we can infer divergence is likely due to *trans*-acting factors (33–36).

We tested 5,898 genes for ASE based on the presence of fixed differences between parental Brazil and New York lines (*Materials and Methods*). While most genes showed conserved gene regulation between New York and Brazil mice (~75%), genes with evidence for expression divergence tended to involve changes in *cis* (Fig. 3*B*). Specifically, 7 to 8% of genes showed expression divergence due to *cis* alone, and 5 to 6% genes showed evidence of divergence due to *cis* and *trans* (Fig. 3*B*). Only ~5% of genes involved regulatory changes solely in *trans* (Fig. 3*B*). Moreover, the magnitude of *cis*-effects was greater than *trans*-effects per gene (Wilcoxon signed-rank tests; BAT, *P* = 2.97 × 10^−27^; liver, *P* = 4.64 × 10^−29^). The predominance of *cis*-regulatory changes relative to *trans*-changes is consistent with previous studies in house mice (37–40).

Genes with evidence for *cis-*divergence were enriched for gene ontology (GO) terms related to metabolic processes, as well as the pathway for metabolism (Reactome R-MMU-1430728; liver, FDR = 6.55 × 10^−8^; BAT, FDR = 1.49 × 10^−8^). In the liver, genes with *cis*-regulatory changes showed a greater than twofold enrichment of genes with mutant phenotypes for abnormal susceptibility to weight gain (FDR = 0.014) and were nominally significantly enriched (with unadjusted *P* values) for several other phenotypes related to body weight, size, and composition (*SI Appendix*, Fig. S6). Interestingly, two genes (*bcat2* and *adam17*) exhibiting *cis*-regulatory divergence were previously implicated in body weight differences in North American populations (24), further supporting their role in adaptive divergence between house mouse populations.

### Most *Cis*-Changes Are Robust to Environmental Temperature.

We next asked how the environment modulates gene regulatory evolution by comparing patterns of *cis*- and *trans*-regulatory differences across environments. Similar to expression patterns observed in the parents, the majority of genes that could be categorized across temperature treatments showed the same regulatory mode in both environments (~88%) (*SI Appendix*, Fig. S7). For the genes that did show a change in regulatory mode, we found that *cis*-regulatory changes were more insensitive to temperature than *trans*-changes (Fig. 3*C*). Comparing the difference in magnitude of the *cis*- and *trans*-differences between warm and cold conditions, we found that *trans*-differences were greater between environments for both tissues (Wilcoxon signed-rank tests, *P* < 2.2 × 10^−16^) (*SI Appendix*, Fig. S8). The cold environment also had a lower proportion of genes with *trans*-divergence (Chi-square tests; BAT, *P* = 0.0003; liver, *P* = 0.02), where the proportion of genes with only *cis*-divergence was the same across temperature conditions (Chi-square tests; BAT, *P* = 0.51; liver, *P* = 0.66). These results indicate that *trans*-effects play a larger role in gene expression plasticity than *cis*-effects.

Given that much of gene expression plasticity is governed by changes in *trans*, we next asked whether the observed correlation between plastic and evolved changes (i.e., Fig. 2*C*) is also seen for genes controlled in *cis*, since expression variation at such genes is not expected to be correlated (41). We therefore compared plastic expression differences with evolved expression differences in genes with evidence for *cis-*regulatory divergence (i.e., ASE) (42). Similar to our previous findings, we found that expression plasticity generally goes in the same direction as evolved divergence in the liver (Spearman’s rho = 0.261, *P* < 0.05) (*SI Appendix*, Fig. S9). However, no correlation was observed in BAT (Spearman’s rho = −0.0374, *P* > 0.05), suggesting that correlated expression patterns in BAT may be regulated by one or a few *trans*-acting modifiers.

### A Small Number of Genes Show Temperature-Dependent *Cis*-Regulation.

While most *cis*-effects were robust to temperature, we were specifically interested in exploring whether any genes showed temperature-dependent *cis*-effects. Such genes are of particular interest since they correspond to plasticity expression quantitative trait loci (*plasticity*-eQTL; i.e., loci that harbor mutations underlying a plastic response) (43). To identify genes for which there was a significant effect of temperature on regulatory divergence, we determined whether either the *cis* and/or the *trans* component showed a significant interaction with temperature (*Materials and Methods*). We identified *cis* x temperature effects for 11 genes in BAT (*gstt1, wars2, hsd11b1, itih5, dst, tmed2, plbd1, cdh13, scd1, tmem45b,* and *s100a13*) and 4 in the liver (*elovl3, hmgcs2, wars2,* and *ebpl*) (FDR < 0.1; 12/15 genes at FDR < 0.05) (*SI Appendix*, Fig. S10). Most of these genes showed differences in the magnitude of ASE between temperatures, but we also observed cases where ASE was induced by one temperature treatment (i.e., *wars2, tmed2, cdh13, s100a13, ebpl,* and *hmgcs2*). Over half of the genes corresponding to *plasticity*-eQTL showed a smaller plastic response in New York than in Brazil, consistent with the overall reduction in expression plasticity in cold-adapted mice. We also identified genes with significant *trans* x temperature effects in BAT (18 genes) and liver (1 gene) (FDR < 0.1; 10/19 genes at FDR < 0.05) (*SI Appendix*, Table S3). Several of these genes with temperature-induced regulatory differences have suggested roles in energy metabolism and thermal tolerance (e.g., refs. 44–47).

### Positive Selection on Genes with *Cis*-Regulatory Divergence in Wild House Mouse Populations.

**Fig. 4.**
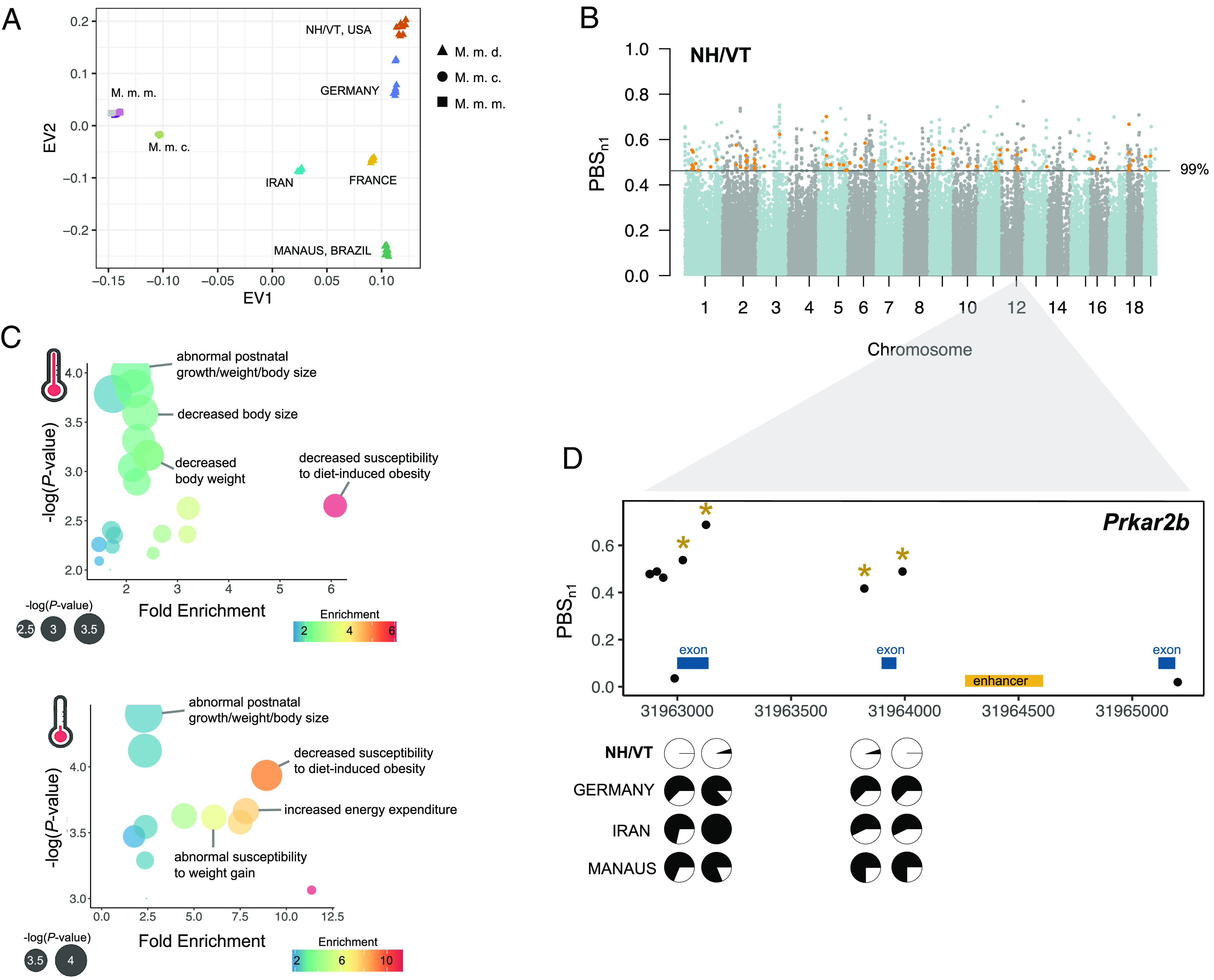
Genomic outliers are enriched in genes with evidence for *cis*-regulatory divergence. (*A*) Genetic PCA of wild house mice distinguished mouse populations based on population-of-origin (M.m.d.) and subspecies (M.m.c.), (M.m.m.). The x and y axes show the first and second SNP eigenvectors, respectively (EV; PC1: 29% of variance, PC2: 8% of variance). (*B*) Autosomal selection scan showing *PBSn1* results for the New Hampshire/Vermont (NH/VT) focal population. Orange points depict genes that exhibit *cis-*regulatory divergence and overlap with outlier regions. (*C*) Gene set enrichment analysis for genes with ASE that overlap genomic outliers in the NH/VT population. ASE outliers were highly enriched for mouse phenotypes related to body size differences and metabolic features, across both temperature treatments. (*D*) Candidate gene that exhibits *cis*-regulatory divergence and overlaps with outlier region. Pie charts depict allele frequencies of four significant SNPs (denoted as gold asterisks) in the four M.m.d. populations.

As *cis*-regulatory variants are often drivers of local adaptation (4, 48, 49), and because most regulatory divergence between New York and Brazil house mice is governed in *cis*, we next explored whether genes regulated in *cis* are under positive selection in wild mice from the Americas. To test this, we used previously published whole exome data from wild-caught individuals collected from New Hampshire/Vermont, USA (NH/VT) (21), and Manaus, Brazil (MAN), and compared these data to previously published whole genome data from Eurasian populations of house mice (50). Genetic PCA distinguished mice based on subspecies and population of origin (Fig. 4*A* and *SI Appendix*, Fig. S11), with mice from NH/VT clustering most closely with mice from Germany. These results support recent findings that mice from eastern North America are most closely related to populations in northern Europe (51–53).

Next, to identify genetic signatures of adaptation in house mice from the Americas, we performed a scan for regions of genetic differentiation consistent with selection using a normalized version of the population branch statistic (PBS). We used this test to identify highly differentiated loci in our focal populations in the Americas (MAN and NH/VT) relative to Eurasian populations (*Materials and Methods*). In total, 83,538 and 84,420 nonoverlapping 5 single nucleotide polymorphism (SNP) windows were analyzed for MAN and NH/VT, respectively. Outlier windows in NH/VT and MAN overlapped 538 and 530 genes, respectively (Dataset S1). Overall, genomic outliers were distributed across the genome in both populations (Fig. 4*B* and *SI Appendix*, Fig. S12), consistent with selection acting primarily on standing genetic variation in house mice (20, 21).

Finally, we asked to what extent genomic divergence among wild mice from temperate and tropical environments is associated with *cis*-regulatory changes. Specifically, if natural selection associated with climatic adaptation has acted mainly on regulatory variants, we predicted an enrichment of genomic outliers near genes exhibiting ASE (e.g., ref. 54). To test this prediction, we overlapped putative candidate regions for selection based on SNP outlier windows with genes for which we identified evidence for ASE in BAT or liver. In NH/VT, we found that outlier windows overlapped 71 and 62 genes with evidence for *cis-*regulatory divergence under warm and cold conditions, respectively (overlap 44 genes) (Fig. 4*B* and Dataset S1). The overlap between genes with *cis-*regulatory divergence and outlier windows in this population was greater than expected by chance (hypergeometric test, *P* = 0.0016) and therefore is unlikely to be a consequence of genetic drift. Moreover, genes with ASE were associated with higher average PBS scores than background genes (*P* = 0.00026, see *Materials and Methods*). In contrast, we did not find significant overlap between genes with ASE and genomic outliers for Manaus (*P* = 0.4). Outlier windows overlapped 49 and 51 genes with evidence for *cis-*regulatory divergence under warm and cold conditions, respectively (*SI Appendix*, Fig. S12). These genes were not enriched for metabolic process terms or phenotypes.

Genes with ASE that overlapped genomic outliers in temperate mice were enriched for mutant phenotypes related to body size, growth, and metabolism relative to other genes with *cis-*regulatory divergence (e.g., abnormal postnatal growth/weight/body size, abnormal susceptibility to weight gain, decreased susceptibility to diet-induced obesity, and increased energy expenditure; FDR < 0.05) (Fig. 4*C* and Dataset S1). This gene set also includes genes whose expression in the liver was previously associated with body mass variation in natural populations of North American house mice (*bcat2, col6a1, col5a2,* and *col3a1*) (21, 24). Additionally, this set included genes implicated in obesity and metabolic phenotypes in humans (e.g., *wrn, plaat3, prkar2b, sulf2,* and *smoc1*) (Fig. 4*D*) (55) and mice (*SI Appendix*, Table S4). Together, these results suggest that selection has acted on *cis-*regulatory genes related to metabolism and body weight in North American mice (24).

## Discussion

Understanding how both genetic and environmental factors influence gene expression divergence is essential to understanding adaptive evolution. Here, we utilized ASE in liver and BAT to characterize *cis* and *trans* changes underlying expression differences between temperate and tropical house mice when reared under warm and cold laboratory environments. We found that most regulatory divergence was governed by *cis*-regulatory variation and that these *cis*-effects were largely independent of environmental temperature. However, a subset of genes showed temperature-dependent *cis*-effects and thus represent QTL for expression plasticity. Finally, overlap of genes exhibiting *cis*-regulatory divergence with scans for selection identified several *cis*-regulatory genes under positive selection, consistent with a role for these loci in local adaptation. Together, our results demonstrate how both genetic and environmental effects contribute to adaptive gene expression differences between natural populations.

The rapid colonization of house mice into new environments may have been mediated by plasticity through *trans*-regulation. Specifically, and similar to previous studies, we found that most expression plasticity was largely governed by *trans*-acting factors (43, 56–62), which modify correlated changes in gene expression profiles of hundreds of genes. In fact, a large proportion of the correlated plastic changes we observed went in the same direction as evolved expression divergence, implicating a role for gene expression plasticity in the colonization of new environments (22). Furthermore, this rapid response via *trans*-effects likely shifted to favor the predominant and less pleiotropic *cis*-regulatory architecture over time (59, 63). We found that most *cis*-effects were robust to temperature, indicating a decoupling of environmental plasticity and allelic-effects. Interestingly, a number of these *cis*-regulatory loci show reduced plasticity in temperate mice (*SI Appendix*, Fig. S13), suggesting that selection may target genetic changes that minimize plasticity (29, 32, 64).

Although most *cis*-effects were robust to temperature, we identified a subset of genes that showed temperature-dependent *cis*-effects. These loci are of particular interest since these constitute *plasticity*-eQTL and harbor mutations that directly affect plasticity of gene expression. Genetic assimilation refers to the conversion of a plastic response to a canalized response (65–68). If the ancestral allele at a *plasticity*-eQTL encodes a plastic response and the derived allele encodes a canalized response, then the *plasticity*-eQTL represents a case of genetic assimilation. For example, selection in a cold, temperate environment may have led to the reduced plasticity exhibited in New York mice. A similar mechanism was recently proposed by Verta and Jones (59) to explain the observed plasticity in expression between freshwater and marine threespine sticklebacks. *Cis*-regulatory variants could rapidly canalize expression through the loss or gain of specific binding sites for conditionally expressed transcription factors, thereby decoupling a gene’s expression from the environment (69). Many of the *cis* x environment candidates illustrate potential regulatory mechanisms underlying genetic assimilation as many of them exhibit reduced plasticity in New York mice (*SI Appendix*, Fig. S13). For example, *scd1* plays an important role in basal and cold-induced thermogenesis (70, 71) and New York mice show higher and constitutive average expression of *scd1* in BAT compared to Brazil mice (*SI Appendix*, Fig. S13). Further study of these genes may help us understand the relationship between plasticity, selection, and adaptation to novel environments in natural populations.

Our comparison between New York and Brazil house mice across environments has implications for our understanding of gene regulation and genome function across short evolutionary timescales. Although house mice colonized the Americas within the last ~500 y, we found evidence for pervasive *cis*-regulatory divergence. Furthermore, house mice have rapidly adapted to various environments from preexisting standing genetic variation (20, 21, 24, 72), which has likely contributed to the predominance and enrichment of *cis*-regulatory variation associated with local adaptation in temperate mice. We speculate that the significant overlap between genomic outliers and ASE in temperate mice but not in tropical mice may reflect adaptation primarily to cold environments (rather than to warm environments), consistent with the warm ancestral range of house mice in the Mediterranean region. Nonetheless, positive selection may preferentially act on *cis*-acting alleles due to their higher additivity and less sensitivity to genomic background (35, 59, 73). Similarly, natural selection may target *cis*-acting alleles due to their insensitivity to environmental conditions, making them a primary substrate for adaptation to novel environments (59). These features of *cis*-regulatory divergence allow them to accrue on extremely short timescales, making them important loci for rapid climatic adaptation.

Overall, this study broadens our understanding of the role of gene regulation in recent adaptive evolution by disentangling *cis*- and *trans*-changes underlying genetic and environmental effects on gene expression differences. While some progress has been made on the relative importance of *cis*- and *trans*-changes in adaptation within and between species (16, 36), most of the observed differences in regulatory patterns have been measured in a single environment, overlooking environment- and genotype-by-environment effects. By pairing ASE across different conditions with genomic data from natural populations, we identified important roles for environment-dependent *trans*-changes and environment-independent *cis*-regulatory divergence in populations adapting to new environments. Thus, this work provides insight into the molecular architecture underlying genetic and nongenetic causes of gene expression differences during adaptive evolution.

## Materials and Methods

### Animals and Evolved Phenotypic Differences.

To characterize evolved phenotypic differences between New York and Brazil house mice, we used two wild-derived inbred lines of house mice: SARA (New York) and MANA (Brazil). Previous studies have demonstrated that these lines vary in morphology and gene expression and are indicative of population divergence (21, 23) (*SI Appendix, Supplementary Text*). The establishment of these lines has been described previously (23). Mice from each line were housed in a standard laboratory environment at 21 °C with a 12L:12D cycle. Roughly equal numbers of males and females were produced for each within-line comparison (*n* = 32 per line; Dataset S1). We took standard museum measurements on all mice and removed and prepared dried skins. Thermal conductance of pelage [referred to as pelage conductance (W m^−2^ °C^−1^)] was measured on dry skins following the protocol of Riddell et al. (74) (*SI Appendix, Supplementary Text*). Tail length and ear length were corrected for body mass for each individual. Effects of line and sex for each phenotype were modeled using ANOVA. All statistical analyses were performed using packages available in R (v.4.1.1).

### Experimental Design and Tissue Collection.

To investigate the gene regulatory mechanisms underlying local adaptation in house mice, we generated F1 hybrids by crossing a SARA female with a MANA male. All experimental animals were born at room temperature (21 °C) and were provided water and commercial rodent chow ad libitum. We weaned and singly housed SARA, MANA, and F1 hybrids at ~3 wk of age. We split 3.5-wk-old full-sibs and F1 hybrids into size-matched experimental groups across cold (5 °C) and warm (21 °C) treatments. Mice were kept in their respective experimental environment until ~12 wk of age. Following euthanasia, we took standard museum measurements and then rapidly dissected and preserved liver and BAT in RNAlater at 4 °C overnight and moved to −80 °C until RNA extraction. We prepared standard museum skeletons and accessioned them in UC Berkeley’s Museum of Vertebrate Zoology (catalog numbers are given in Dataset S1). All experimental procedures were in accordance with the UC Berkeley Institutional Animal Care and Use Committee (AUP-2017-08-10248).

### RNA Extraction, Library Preparation, and Sequencing.

We extracted total RNA from liver and BAT from each sample (*n* = ~6 per genotype/sex/treatment/tissue) using the Rneasy PowerLyzer Kit (QIAGEN). We generated Illumina cDNA libraries from 1 µg of purified RNA using KAPA Stranded mRNA-Seq Kit (Illumina) and uniquely indexed libraries using unique dual indexes (Illumina). Libraries were pooled in equal molar concentration and sequenced on one lane each of 150 bp paired-end NovaSeq S1 and NovaSeq S4 at the Vincent J. Coates Genomics Sequencing Center at UC Berkeley. We filtered raw reads below a Phred quality score of 15 and trimmed adapter sequences using *fastp* (75).

### Parental Gene Expression Analyses.

After cleaning and trimming parental sequences of MANA and SARA, we mapped reads to the *Mus musculus* reference genome (GRCm38/mm10) using STAR (76). We counted reads overlapping exons using HTSeq (77) based on the Ensembl GRCm38.98 annotation. We imported raw count data into R (v.4.1.1) and transformed expression values using variance stabilizing transformation (78) to assess transcriptome-wide expression patterns via PCA. Next, we removed genes with fewer than an average of 10 reads per individual within each tissue, retaining ~14K expressed genes per tissue for downstream analyses. We then used DESeq2 (78) on raw, filtered reads to quantify expression patterns by fitting a generalized linear model following a negative binomial distribution (Wald-test). Due to the strong effects of tissue type and sex on expression patterns (*SI Appendix*, Fig. S1), we computed differential expression between lines for each tissue and sex, separately (but see *SI Appendix, Supplementary Text*, for a full parameterized model). Specifically, we used the model line + environment + line*environment to determine the effects of genotype, environment, and genotype-by-environment (GxE) on expression patterns. We defined genes as GxE if: 1) only one genotype showed significant differential expression between temperatures (“line-specific”), or 2) both genotypes showed significant differences between temperatures, but in opposite directions (“opposite”). Last, we used a Benjamini–Hochberg multiple test correction (79) on all resulting *P* values and considered genes with FDR < 0.05 to be significantly differentially expressed.

To determine whether gene expression plasticity is correlated with gene expression divergence, we compared genes with significant plasticity in Brazil mice to genes with significant expression divergence between Brazil and New York mice within each tissue and sex, separately. We used Spearman’s rank correlation coefficients to assess overall directionality and significance of gene expression. Since this comparison involves two ratios that share the same denominator, we ensured that the observed patterns were not a consequence of spurious correlations by randomly choosing two different sets of three replicates of warm Brazil mice for each comparison [e.g., warm New York vs. warm Brazil samples 1-3 (*y* axis) and cold Brazil vs. warm Brazil samples 4-6 (*x* axis)]. All significant correlations were maintained within each tissue and sex (positive Spearman's correlations, *P* < 0.05). We also compared the observed correlations to a permuted distribution (10,000 permutations) to account for potential statistical artifacts in the regression (80).

### Identifying Variants between Parental Lines.

To identify differences between lines for allele-specific read assignment, we performed SNP calling on whole genome sequence data from one female each of MANA and SARA. We mapped genomic reads with Bowtie2 (81) to the mm10 reference genome (setting: –very-sensitive) obtained from Ensembl. We marked duplicates with the Picard tool MarkDuplicates, and then we used the Genome Analysis Toolkit (GATK) tools HaplotypeCaller and GenotypeGVCFs for joint genotyping across genomic samples. We filtered for low-quality SNP calls with VariantFiltration (QD < 2.0; QUAL < 30.0; FS > 200; ReadPosRankSum < −20.0). To reduce the influence of genotyping error in whole-genome sequencing data on ASE assignment of RNA-seq reads (e.g., ref. 82), we mapped RNA-seq reads from all individuals and then counted allele-specific reads aligned to each site we genotyped with the GATK tool ASEReadCounter. We excluded sites for which we did not have coverage of at least 5 reads from each population-specific allele. In total, 2,875,480 and 2,181,304 variants from MANA and SARA, respectively, were used for identifying allele-specific reads.

### Mapping Allele-Specific Reads.

For ASE analyses, we mapped reads from hybrid individuals to the mouse reference genome (GRCm38/mm10) using STAR. We used WASP (83) to reduce the potential for reference mapping bias. Specifically, WASP mitigates mapping bias by identifying reads containing SNPs, simulating reads with alternative alleles at that locus, remapping these reads to the reference, and then flagging reads that do not map to the same location. Reads that do not map to the same location are then discarded. We retained reads that overlapped a population-specific variant and that passed WASP filtering for our ASE analysis. We separated reads overlapping informative variants into allele-specific pools (NY, BZ) based on genotype for quantification. We used HTSeq to count the number of reads associated with each gene per population based on the overlap of reads and annotated exonic regions based on the Ensembl GRCm38.98 annotation. We examined per site allelic reads with ASEReadCounter to quantify allele-specific mapping over individual sites. Proportions of reads overlapping the references vs. alternative allele [REF allele/(ALT allele + REF allele)] showed a median 0.5 across samples (*SI Appendix*, Figs. S14 and S15), indicating no evidence for reference mapping bias.

### Identifying *Cis*- and *Trans*-Regulatory Divergence.

Parental (F0) and F1 expression data were used to characterize *cis* and *trans* effects. To categorize regulatory divergence at each gene, we inferred differential expression by analyzing raw counts using DESeq2. To identify genes with evidence of allele-specific expression in hybrid individuals, we took reads that mapped preferentially to either New York or Brazil alleles and fit these to a model with allele (NY vs. BZ), sample (individual), and tissue (BAT, liver) for hybrid male samples in DESeq2 (Wald test; *SI Appendix, Supplementary Text*). As read counts come from the same sequencing library, library size factor normalization was disabled in DESeq2 by setting SizeFactors = 1 for measures of allele-specific expression. We used males to assign regulatory categories to maximize power due to a larger number of hybrid samples sequenced (6 replicates of males vs. 4 replicates of females), although we also see similar regulatory patterns in females (*SI Appendix, Supplementary Text* and Fig. S16). Differential expression between alleles in the F1 is evidence for *cis-*regulatory divergence. Conversely, *trans*-regulatory divergence is inferred when differential expression in the F0 generation is not recapitulated between alleles in the F1. The *trans* component (*T*) was assessed through a Fisher’s exact test on reads mapping to each parental allele in the hybrid vs. parental read counts, summed over all replicates (35, 84). We randomly down-sampled reads to account for library size differences between parental and F1 replicates (39, 85). *P*-values for each test were corrected for FDR with the Benjamini–Hochberg method. We sorted genes into categories based on an FDR threshold of 5% (35, 84), as described below. We analyzed temperature treatments (warm and cold) separately for regulatory assignment and then compared as described below:

*Conserved:* no significant difference between lines (F0), no significant difference between alleles (F1), no significant *T*.
*Cis only:* significant difference between lines (F0), significant difference between alleles (F1), no significant *T*.
*Trans only:* significant difference between lines (F0), no significant difference between alleles (F1), significant *T*.
*Cis* & *Trans* designations: significant differences between alleles (F1) and significant *T*. This category was further subdivided into *cis* + *trans* (reinforcing), *cis* + *trans* (opposing), *compensatory*, and *cis* x *trans*, as previously described (37, 39).
*Ambiguous*: all other patterns

We identified *cis* x temperature interactions using DESeq2 under a model specifying temperature (cold vs. warm) and allele (BZ vs. NY). To identify *trans* x temperature interactions, we fit a model that included parental and hybrid read counts for temperature (cold vs. warm), allele/genotype (BZ vs. NY), generation (F1 vs. F0), and interactions. We considered genes significant at an FDR threshold of 10%, consistent with previous studies (e.g., refs. 59 and 86) as our statistical analysis has less power to detect interactions than main effects. Similar models were also used to identify sex- and tissue-specific regulatory patterns in DESeq2 (*SI Appendix, Supplementary Text*).

### Genetic PCA of M.m.d. Populations.

We used SNPRelate (87) to perform PCA and IBS hierarchical clustering of population genetic data. Genomic data from 3 Eurasian populations of M.m.d. [Germany (Cologne-Bonn), France, and Iran] and *Mus musculus musculus* (M.m.m.) *and Mus musculus castaneus* (M.m.c.) subspecies were downloaded from http://wwwuser.gwdg.de/~evolbio/evolgen/wildmouse/ (50). For PCA, biallelic variants genotyped across all these individuals were extracted and pruned for linkage disequilibrium in SNPRelate (thresholds = 0.2) resulting in 22,126 variant sites for PCA and IBS clustering for M.m.d. comparisons and 25,467 variants for global *Mus* comparisons (Fig. 4*A* and *SI Appendix*, Fig. S11). Altering the pruning threshold to 0.5 did not result in any change in population clustering.

### Autosomal Scans for Selection.

To identify regions with evidence for selection in the Americas, we scanned the exomes of our North and South American focal populations for selection by using a modification of the PBS which summarizes a three-way comparison of allele frequencies between a focal group, a closely related population, and an outgroup comparison (*PBSn1*) (88, 89):

$$PBSn1=\frac{{\mathrm{PBS}}_{1}}{1+{\mathrm{PBS}}_{1}+{\mathrm{PBS}}_{2}+{\mathrm{PBS}}_{3}}.$$

Here, *PBS_1_* indicates PBS calculated as either Manaus or NH/VT as the focal population, and *PBS_2_* and *PBS_3_* indicate PBS calculated for Eurasian populations as the focal populations (France or Germany and Iran, respectively). To maximize the number of sites that could be compared, American populations are not directly compared in the branch test due to the reduced representation of exome data and high per site Fst values between the two populations (*SI Appendix*, Fig. S17). Instead, NH/VT and MAN were each compared to two Eurasian populations [((MAN), France) Iran) and ((NH/VT) Germany) Iran)], selected based on population clustering (*SI Appendix*, Fig. S11). We restricted our SNP set to biallelic variants across the 3 populations being compared and required that at least six individuals in the focal branch be genotyped. We note that the NH/VT sample used in the PBS test is geographically close to the origin of the SARA line.

We used VCFtools (90) to calculate Weir and Cockerham Fst at each variant position. These values were used to calculate *PBSn1* for nonoverlapping blocks of 5 SNPs. We consider blocks in the top 1% of *PBSn1* scores outliers and do not attempt to assign *P* values to each SNP block (91). Genomic outliers were >3 SDs above the mean windowed value of SNP blocks in each comparison (MAN focal, median = 0.045; NH/VT focal median = 0.064). We refer to these loci as “genomic outliers” given the selection scan did not consider neutral models of evolution. We identified windows overlapping genes based on Ensembl gene coordinates (mm10) and the BEDTools “intersect” tool (92). As allele-specific expression in F1s is consistent with local independent genetic changes influencing gene expression, we focused on genes with evidence for *cis-*regulatory divergence (i.e., differences in expression between parental alleles in the F1) for overlap with outlier loci. To ask whether allele-specific expression was associated with elevated *PBSn1* scores, we used a generalized linear model incorporating gene category (ASE or no ASE) and SNP density per kb as factors to *PBSn1* scores. SNP density was calculated by dividing the number of informative sites between NY and BZ for allele-specific expression per gene by transcript length.

### Enrichment Analyses.

We performed all GO and pathway enrichment analyses with PANTHER (93, 94). For GO enrichment associated with *cis*-regulatory divergence, we defined the background set of genes as all *cis*-regulated genes tested within a tissue. We performed phenotype enrichment analyses with ModPhea (95), and we annotated genes to specific phenotypes based on Mouse Genome Informatics phenotype annotations (http://www.informatics.jax.org/).

## Supplementary Material

Appendix 01 (PDF)

Dataset S01 (XLSX)

## Data Availability

Scripts are available on GitHub (96), with the repository archived in Zenodo (97). All sequence data generated in this study have been deposited to the National Center for Biotechnology Information Sequence Read Archive under accession BioProject ID PRJNA1009445 (98). All other data are included in the article and/or supporting information.

## References

[1] M. C. King, A. C. Wilson, Evolution at two levels in humans and chimpanzees. Science **188**, 107–116 (1975). 1090005 10.1126/science.1090005

[2] G. A. Wray , The evolution of transcriptional regulation in eukaryotes. Mol. Biol. Evol. **20**, 1377–1419 (2003). 12777501 10.1093/molbev/msg140

[3] F. C. Jones , The genomic basis of adaptive evolution in threespine sticklebacks. Nature **484**, 55–61 (2012). 22481358 10.1038/nature10944 PMC3322419

[4] H. B. Fraser, Gene expression drives local adaptation in humans. Genome Res. **23**, 1089–1096 (2013). 23539138 10.1101/gr.152710.112 PMC3698502

[5] G. Gibson, The environmental contribution to gene expression profiles. Nat. Rev. Genet. **9**, 575–581 (2008). 18574472 10.1038/nrg2383

[6] V. Grishkevich, I. Yanai, The genomic determinants of genotype× environment interactions in gene expression. Trends Genet. **29**, 479–487 (2013). 23769209 10.1016/j.tig.2013.05.006

[7] A. Hodgins-Davis, J. P. Townsend, Evolving gene expression: From G to E to GxE. Trends Ecol. Evol. **24**, 649–658 (2009). 19699549 10.1016/j.tree.2009.06.011 PMC2805859

[8] L. López-Maury, S. Marguerat, J. Bähler, Tuning gene expression to changing environments: From rapid responses to evolutionary adaptation. Nat. Rev. Genet. **9**, 583–593 (2008). 18591982 10.1038/nrg2398

[9] M. J. West-Eberhard, Developmental Plasticity and Evolution (Oxford University Press, 2003).

[10] C. K. Ghalambor, J. K. McKAY, S. P. Carroll, D. N. Reznick, Adaptive versus non-adaptive phenotypic plasticity and the potential for contemporary adaptation in new environments. Funct. Ecol. **21**, 394–407 (2007).

[11] A. Corl , The genetic basis of adaptation following plastic changes in coloration in a novel environment. Curr. Biol. **28**, 2970–2977.e7 (2018). 30197088 10.1016/j.cub.2018.06.075

[12] G. A. Wray, The evolutionary significance of *cis*-regulatory mutations. Nat. Rev. Genet. **8**, 206–216 (2007). 17304246 10.1038/nrg2063

[13] P. J. Wittkopp, G. Kalay, *Cis*-regulatory elements: Molecular mechanisms and evolutionary processes underlying divergence. Nat. Rev. Genet. **13**, 59–69 (2011). 22143240 10.1038/nrg3095

[14] S. B. Carroll, Evo-devo and an expanding evolutionary synthesis: A genetic theory of morphological evolution. Cell **134**, 25–36 (2008). 18614008 10.1016/j.cell.2008.06.030

[15] D. L. Stern, V. Orgogozo, The loci of evolution: How predictable is genetic evolution? Evolution **62**, 2155–2177 (2008). 18616572 10.1111/j.1558-5646.2008.00450.x PMC2613234

[16] S. A. Signor, S. V. Nuzhdin, The evolution of gene expression in *cis* and *trans*. Trends Genet. **34**, 532–544 (2018). 29680748 10.1016/j.tig.2018.03.007 PMC6094946

[17] T. F. Cooper, D. E. Rozen, R. E. Lenski, Parallel changes in gene expression after 20,000 generations of evolution in *Escherichia coli*. Proc. Natl. Acad. Sci. U.S.A. **100**, 1072–1077 (2003). 12538876 10.1073/pnas.0334340100 PMC298728

[18] D. L. Stern, V. Orgogozo, Is genetic evolution predictable? Science **323**, 746–751 (2009). 19197055 10.1126/science.1158997 PMC3184636

[19] C. B. Lynch, Clinal variation in cold adaptation in *Mus domesticus*: Verification of predictions from laboratory populations. Am. Nat. **139**, 1219–1236 (1992).

[20] K. G. Ferris , The genomics of rapid climatic adaptation and parallel evolution in North American house mice. PLoS Genet. **17**, e1009495 (2021). 33914747 10.1371/journal.pgen.1009495 PMC8084166

[21] M. Phifer-Rixey , The genomic basis of environmental adaptation in house mice. PLoS Genet. **14**, e1007672 (2018). 30248095 10.1371/journal.pgen.1007672 PMC6171964

[22] N. K. J. Bittner, K. L. Mack, M. W. Nachman, Gene expression plasticity and desert adaptation in house mice. Evolution **75**, 1477–1491 (2021). 33458812 10.1111/evo.14172 PMC8218737

[23] M. A. Ballinger, M. W. Nachman, The contribution of genetic and environmental effects to Bergmann’s rule and Allen’s rule in house mice. Am. Nat. **199**, 691–704 (2022). 35472023 10.1086/719028

[24] K. L. Mack, M. A. Ballinger, M. Phifer-Rixey, M. W. Nachman, Gene regulation underlies environmental adaptation in house mice. Genome Res. **28**, 1636–1645 (2018). 30194096 10.1101/gr.238998.118 PMC6211637

[25] N. A. Abumrad, The liver as a hub in thermogenesis. Cell Metab. **26**, 454–455 (2017). 28877451 10.1016/j.cmet.2017.08.018

[26] J. Simcox , Global analysis of plasma lipids identifies liver-derived acylcarnitines as a fuel source for brown fat thermogenesis. Cell Metab. **26**, 509–522.e6 (2017). 28877455 10.1016/j.cmet.2017.08.006 PMC5658052

[27] B. Cannon, J. Nedergaard, Brown adipose tissue: Function and physiological significance. Physiol. Rev. **84**, 277–359 (2004). 14715917 10.1152/physrev.00015.2003

[28] T. D. Price, A. Qvarnström, D. E. Irwin, The role of phenotypic plasticity in driving genetic evolution. Proc. Biol. Sci. **270**, 1433–1440 (2003). 12965006 10.1098/rspb.2003.2372 PMC1691402

[29] E. K. Fischer, C. K. Ghalambor, K. L. Hoke, Can a network approach resolve how adaptive vs nonadaptive plasticity impacts evolutionary trajectories? Integr. Comp. Biol. **56**, 877–888 (2016). 27400976 10.1093/icb/icw087

[30] C. K. Ghalambor , Non-adaptive plasticity potentiates rapid adaptive evolution of gene expression in nature. Nature **525**, 372–375 (2015). 26331546 10.1038/nature15256

[31] E. B. Josephs, M. L. Van Etten, A. Harkess, A. Platts, R. S. Baucom, Adaptive and maladaptive expression plasticity underlying herbicide resistance in an agricultural weed. Evol. Lett. **5**, 432–440 (2021). 34367667 10.1002/evl3.241 PMC8327940

[32] S. C. Campbell-Staton, J. P. Velotta, K. M. Winchell, Selection on adaptive and maladaptive gene expression plasticity during thermal adaptation to urban heat islands. Nat. Commun. **12**, 6195 (2021). 34702827 10.1038/s41467-021-26334-4 PMC8548502

[33] C. R. Cowles, J. N. Hirschhorn, D. Altshuler, E. S. Lander, Detection of regulatory variation in mouse genes. Nat. Genet. **32**, 432–437 (2002). 12410233 10.1038/ng992

[34] P. J. Wittkopp, B. K. Haerum, A. G. Clark, Evolutionary changes in *cis* and *trans* gene regulation. Nature **430**, 85–88 (2004). 15229602 10.1038/nature02698

[35] C. J. McManus , Regulatory divergence in Drosophila revealed by mRNA-seq. Genome Res. **20**, 816–825 (2010). 20354124 10.1101/gr.102491.109 PMC2877578

[36] M. S. Hill, P. V. Zande, P. J. Wittkopp, Molecular and evolutionary processes generating variation in gene expression. Nat. Rev. Genet. **22**, 203–215 (2020). 33268840 10.1038/s41576-020-00304-w PMC7981258

[37] A. Goncalves , Extensive compensatory *cis*-*trans* regulation in the evolution of mouse gene expression. Genome Res. **22**, 2376–2384 (2012). 22919075 10.1101/gr.142281.112 PMC3514667

[38] S. Q. Shen, E. Turro, J. C. Corbo, Hybrid mice reveal parent-of-origin and *cis*- and *trans*-regulatory effects in the retina. PLoS One **9**, e109382 (2014). 25340786 10.1371/journal.pone.0109382 PMC4207689

[39] K. L. Mack, P. Campbell, M. W. Nachman, Gene regulation and speciation in house mice. Genome Res. **26**, 451–461 (2016). 26833790 10.1101/gr.195743.115 PMC4817769

[40] J. J. Crowley , Analyses of allele-specific gene expression in highly divergent mouse crosses identifies pervasive allelic imbalance. Nat. Genet. **47**, 353–360 (2015). 25730764 10.1038/ng.3222 PMC4380817

[41] J. van Gestel, F. J. Weissing, Is plasticity caused by single genes? Nature **555**, E19–E20 (2018). 29595768 10.1038/nature25495

[42] F. He , *Cis*-regulatory evolution spotlights species differences in the adaptive potential of gene expression plasticity. Nat. Commun. **12**, 3376 (2021). 34099660 10.1038/s41467-021-23558-2 PMC8184852

[43] Y. Li , Mapping determinants of gene expression plasticity by genetical genomics in C. elegans. PLoS Genet. **2**, e222 (2006). 17196041 10.1371/journal.pgen.0020222 PMC1756913

[44] M. Fumagalli , Greenlandic Inuit show genetic signatures of diet and climate adaptation. Science **349**, 1343–1347 (2015). 26383953 10.1126/science.aab2319

[45] B. Hallmark , Genomic evidence of local adaptation to climate and diet in Indigenous Siberians. Mol. Biol. Evol. **36**, 315–327 (2019). 30428071 10.1093/molbev/msy211

[46] A. M. Shore , Cold-induced changes in gene expression in brown adipose tissue, white adipose tissue and liver. PLoS One **8**, e68933 (2013). 23894377 10.1371/journal.pone.0068933 PMC3718809

[47] R. Westerberg , ELOVL3 is an important component for early onset of lipid recruitment in brown adipose tissue. J. Biol. Chem. **281**, 4958–4968 (2006). 16326704 10.1074/jbc.M511588200

[48] H. B. Fraser, A. M. Moses, E. E. Schadt, Evidence for widespread adaptive evolution of gene expression in budding yeast. Proc. Natl. Acad. Sci. U.S.A. **107**, 2977–2982 (2010). 20133628 10.1073/pnas.0912245107 PMC2840270

[49] H. B. Fraser , Systematic detection of polygenic *cis*-regulatory evolution. PLoS Genet. **7**, e1002023 (2011). 21483757 10.1371/journal.pgen.1002023 PMC3069120

[50] B. Harr , Genomic resources for wild populations of the house mouse, *Mus musculus* and its close relative *Mus spretus*. Sci. Data **3**, 160075 (2016). 27622383 10.1038/sdata.2016.75 PMC5020872

[51] H. Tichy, Z. Zaleska-Rutczynska, C. O’Huigin, F. Figueroa, J. Klein, Origin of the North American house mouse. Folia Biol. **40**, 483–496 (1994). 7589706

[52] A. P. Morgan , Population structure and inbreeding in wild house mice (Mus musculus) at different geographic scales. Heredity **129**, 183–194 (2022). 35764696 10.1038/s41437-022-00551-z PMC9411160

[53] K. D. Agwamba, M. W. Nachman, The demographic history of house mice (Mus musculus domesticus) in eastern North America. G3 (Bethesda) **13**, jkac332 (2023). 36546306 10.1093/g3journal/jkac332 PMC9911051

[54] R. A. York , Behavior-dependent *cis* regulation reveals genes and pathways associated with bower building in cichlid fishes. Proc. Natl. Acad. Sci. U.S.A. **115**, E11081–E11090 (2018). 30397142 10.1073/pnas.1810140115 PMC6255178

[55] J. Bérubé, C. Garand, G. Lettre, M. Lebel, The non-synonymous polymorphism at position 114 of the WRN protein affects cholesterol efflux in vitro and correlates with cholesterol levels in vivo. Exp. Gerontol. **48**, 533–538 (2013). 23523974 10.1016/j.exger.2013.03.003

[56] E. N. Smith, L. Kruglyak, Gene-environment interaction in yeast gene expression. PLoS Biol. **6**, e83 (2008). 18416601 10.1371/journal.pbio.0060083 PMC2292755

[57] I. Tirosh, S. Reikhav, A. A. Levy, N. Barkai, A yeast hybrid provides insight into the evolution of gene expression regulation. Science **324**, 659–662 (2009). 19407207 10.1126/science.1169766

[58] J. Chen, V. Nolte, C. Schlötterer, Temperature stress mediates decanalization and dominance of gene expression in *Drosophila melanogaster*. PLoS Genet. **11**, e1004883 (2015). 25719753 10.1371/journal.pgen.1004883 PMC4342254

[59] J.-P. Verta, F. C. Jones, Predominance of *cis*-regulatory changes in parallel expression divergence of sticklebacks. Elife **8**, e43785 (2019). 31090544 10.7554/eLife.43785 PMC6550882

[60] X. C. Li, J. C. Fay, *Cis*-regulatory divergence in gene expression between two thermally divergent yeast species. Genome Biol. Evol. **9**, 1120–1129 (2017). 28431042 10.1093/gbe/evx072 PMC5554586

[61] D. Promislow, A regulatory network analysis of phenotypic plasticity in yeast. Am. Nat. **165**, 515–523 (2005). 15795849 10.1086/429161

[62] S. D. Ding , Trans-regulatory changes underpin the evolution of the Drosophila immune response. PLoS Genet. **18**, e1010453 (2022). 36342922 10.1371/journal.pgen.1010453 PMC9671443

[63] P. Vande Zande, P. J. Wittkopp, Network topology can explain differences in pleiotropy between *cis*- and *trans*-regulatory mutations. Mol. Biol. Evol. **39**, msac266 (2022). 36508350 10.1093/molbev/msac266 PMC9791367

[64] W.-C. Ho, J. Zhang, Genetic gene expression changes during environmental adaptations tend to reverse plastic changes even after the correction for statistical nonindependence. Mol. Biol. Evol. **36**, 604–612 (2019). 30649427 10.1093/molbev/msz002 PMC6657441

[65] C. H. Waddington, Canalization of development and the inheritance of acquired characters. Nature **150**, 563–565 (1942).

[66] C. H. Waddington, Selection of the genetic basis for an acquired character. Nature **169**, 625–626 (1952). 10.1038/169625b0 14929255

[67] C. H. Waddington, Genetic assimilation of an acquired character. Evolution **7**, 118–126 (1953).

[68] K. R. L. van der Burg , Genomic architecture of a genetically assimilated seasonal color pattern. Science **370**, 721–725 (2020). 33154142 10.1126/science.aaz3017

[69] I. M. Ehrenreich, D. W. Pfennig, Genetic assimilation: A review of its potential proximate causes and evolutionary consequences. Ann. Bot. **117**, 769–779 (2016). 26359425 10.1093/aob/mcv130 PMC4845796

[70] J. M. Ntambi , Loss of stearoyl-CoA desaturase-1 function protects mice against adiposity. Proc. Natl. Acad. Sci. U.S.A. **99**, 11482–11486 (2002). 12177411 10.1073/pnas.132384699 PMC123282

[71] S.-H. Lee , Lack of stearoyl-CoA desaturase 1 upregulates basal thermogenesis but causes hypothermia in a cold environment. J. Lipid Res. **45**, 1674–1682 (2004). 15210843 10.1194/jlr.M400039-JLR200

[72] E. J. Beckman , The genomic basis of high-elevation adaptation in wild house mice (Mus musculus domesticus) from South America. Genetics **220**, iyab226 (2022). 34897431 10.1093/genetics/iyab226 PMC9097263

[73] B. Lemos, L. O. Araripe, P. Fontanillas, D. L. Hartl, Dominance and the evolutionary accumulation of *cis*- and *trans*-effects on gene expression. Proc. Natl. Acad. Sci. U.S.A. **105**, 14471–14476 (2008). 18791071 10.1073/pnas.0805160105 PMC2567206

[74] E. A. Riddell, J. L. Patton, S. R. Beissinger, Thermal adaptation of pelage in desert rodents balances cooling and insulation. Evolution **76**, 3001–3013 (2022). 36221218 10.1111/evo.14643 PMC10091991

[75] S. Chen, Y. Zhou, Y. Chen, J. Gu, fastp: An ultra-fast all-in-one FASTQ preprocessor. Bioinformatics **34**, i884–i890 (2018). 30423086 10.1093/bioinformatics/bty560 PMC6129281

[76] A. Dobin , STAR: Ultrafast universal RNA-seq aligner. Bioinformatics **29**, 15–21 (2013). 23104886 10.1093/bioinformatics/bts635 PMC3530905

[77] S. Anders, P. T. Pyl, W. Huber, HTSeq–a Python framework to work with high-throughput sequencing data. Bioinformatics **31**, 166–169 (2015). 25260700 10.1093/bioinformatics/btu638 PMC4287950

[78] M. I. Love, W. Huber, S. Anders, Moderated estimation of fold change and dispersion for RNA-seq data with DESeq2. Genome Biol. **15**, 550 (2014). 25516281 10.1186/s13059-014-0550-8 PMC4302049

[79] Y. Benjamini, Y. Hochberg, Controlling the false discovery rate: A practical and powerful approach to multiple testing. J. R. Stat. Soc. Series B Stat. Methodol. **57**, 289–300 (1995).

[80] F. Mallard, A. M. Jakšić, C. Schlötterer, Contesting the evidence for non-adaptive plasticity. Nature **555**, E21–E22 (2018). 29595765 10.1038/nature25496

[81] B. Langmead, S. L. Salzberg, Fast gapped-read alignment with Bowtie 2. Nat. Methods **9**, 357–359 (2012). 22388286 10.1038/nmeth.1923 PMC3322381

[82] L. Frésard , Identification of rare-disease genes using blood transcriptome sequencing and large control cohorts. Nat. Med. **25**, 911–919 (2019). 31160820 10.1038/s41591-019-0457-8 PMC6634302

[83] B. van de Geijn, G. McVicker, Y. Gilad, J. K. Pritchard, WASP: Allele-specific software for robust molecular quantitative trait locus discovery. Nat. Methods **12**, 1061–1063 (2015). 26366987 10.1038/nmeth.3582 PMC4626402

[84] J. D. Coolon, C. J. McManus, K. R. Stevenson, B. R. Graveley, P. J. Wittkopp, Tempo and mode of regulatory evolution in Drosophila. Genome Res. **24**, 797–808 (2014). 24567308 10.1101/gr.163014.113 PMC4009609

[85] J. D. Coolon, C. J. McManus, K. R. Stevenson, B. R. Graveley, P. J. Wittkopp, Corrigendum: Tempo and mode of regulatory evolution in Drosophila. Genome Res. **28**, 1766 (2018). 30385616 10.1101/gr.244087.118 PMC6211639

[86] K. Mattioli , *Cis* and *trans* effects differentially contribute to the evolution of promoters and enhancers. Genome Biol. **21**, 210 (2020). 32819422 10.1186/s13059-020-02110-3 PMC7439725

[87] X. Zheng , A high-performance computing toolset for relatedness and principal component analysis of SNP data. Bioinformatics **28**, 3326–3328 (2012). 23060615 10.1093/bioinformatics/bts606 PMC3519454

[88] X. Yi , Sequencing of 50 human exomes reveals adaptation to high altitude. Science **329**, 75–78 (2010). 20595611 10.1126/science.1190371 PMC3711608

[89] J. E. Crawford , Natural selection on genes related to cardiovascular health in high-altitude adapted Andeans. Am. J. Hum. Genet. **101**, 752–767 (2017). 29100088 10.1016/j.ajhg.2017.09.023 PMC5673686

[90] P. Danecek , The variant call format and VCFtools. Bioinformatics **27**, 2156–2158 (2011). 21653522 10.1093/bioinformatics/btr330 PMC3137218

[91] A.-S. Malaspinas , A genomic history of Aboriginal Australia. Nature **538**, 207–214 (2016). 27654914 10.1038/nature18299 PMC7617037

[92] A. R. Quinlan, I. M. Hall, BEDTools: A flexible suite of utilities for comparing genomic features. Bioinformatics **26**, 841–842 (2010). 20110278 10.1093/bioinformatics/btq033 PMC2832824

[93] H. Mi, A. Muruganujan, P. D. Thomas, PANTHER in 2013: Modeling the evolution of gene function, and other gene attributes, in the context of phylogenetic trees. Nucleic Acids Res. **41**, D377–86 (2013). 23193289 10.1093/nar/gks1118 PMC3531194

[94] P. D. Thomas , PANTHER: A library of protein families and subfamilies indexed by function. Genome Res. **13**, 2129–2141 (2003). 12952881 10.1101/gr.772403 PMC403709

[95] M.-P. Weng, B.-Y. Liao, modPhEA: Model organism phenotype enrichment analysis of eukaryotic gene sets. Bioinformatics **33**, 3505–3507 (2017). 28666356 10.1093/bioinformatics/btx426

[96] M. A. Ballinger, Data from “Environmentally robust *cis*-regulatory changes underlie rapid climatic adaptation.” Github. https://github.com/malballinger/BallingerMack_NYBZase_2023. Accessed 25 August 2023. 10.1073/pnas.2214614120 PMC10523592 37725649

[97] M. A. Ballinger, Data from “Environmentally robust *cis*-regulatory changes underlie rapid climatic adaptation.” Zenodo. 10.5281/zenodo.8288001. Accessed 25 August 2023. PMC10523592 37725649

[98] M. A. Ballinger, Gene regulatory evolution in temperate and tropical house mice. NCBI BioProject. https://www.ncbi.nlm.nih.gov/bioproject/PRJNA1009445/. Accessed 25 August 2023.

